# Adenoidectomy in a child with Crouzon syndrome complicated with severe obstructive sleep apnea: Case report and review of literature

**DOI:** 10.1097/MD.0000000000038534

**Published:** 2024-06-07

**Authors:** Lei Yu, Yuliang Zhao

**Affiliations:** a Department of Otolaryngology, Second Hospital of Hebei Medical University, Shijiazhuang, China.

**Keywords:** adenoidectomy, Crouzon syndrome, severe obstructive sleep apnea

## Abstract

**Rationale:**

Crouzon syndrome is an extremely rare craniofacial dysplasia, which is mainly caused by the early ossification and closure of the coronal suture of the skull. Craniofacial deformities can cause stenosis of the nasal cavity and posterior nasal meatus, resulting in sleep apnea.

**Patient concerns:**

A 9-year-old boy with sleep snoring for 6 years, progressive aggravation in the past 1 month and accompanied by apnea during sleep.

**Diagnoses:**

This case was diagnosed with Crouzon syndrome complicated with severe obstructive sleep apnea and severe hypoxemia.

**Interventions:**

After adenoidectomy, he was admitted to the pediatric intensive care unit with ventilator-assisted respiration. During this period, the blood oxygen saturation fluctuated greatly. After trying to extubate, the blood oxygen was difficult to maintain and had to be intubated again. After active treatment, extubation was successful.

**Outcomes:**

The wound of nasopharynx recovered well and the sleep state was significantly improved 3 months postoperation.

**Lessons:**

It is suggested that the time of ventilator-assisted breathing should be prolonged and the perioperative airway management should be strengthened in order to reduce the risk of postoperative complications.

## 1. Introduction

Crouzon syndrome is a kind of craniofacial dysplasia. The incidence rate in newborn alive infants is about 1/25,000 and in the general population is about 16/1 million, and a male-female incidence ratio of 3:1.^[1,2]^ The main clinical manifestations of the disease are cranial deformities, facial deformities, exophthalmos, strabismus and orbital widening deformities, and some patients are accompanied by other complications, such as intracranial hypertension, hydrocephalus, hearing loss, nasal deviation, posterior nostril and nasopharyngeal stenosis or atresia, etc., resulting in obstructive sleep apnea and intelectual disability. Therefore, the diagnosis and treatment of the disease require the cooperation of multiple disciplines included pediatrics, ophthalmology, otorhinolaryngology, stomatology, neurosurgery, plastic surgery and other disciplines.^[3,4]^ A case of Crouzon syndrome with severe obstructive sleep apnea and severe hypoxemia was recently treated in our department. The condition, diagnosis and treatment are reported and analyzed as follows.

## 2. Case report

**Figure 1.**
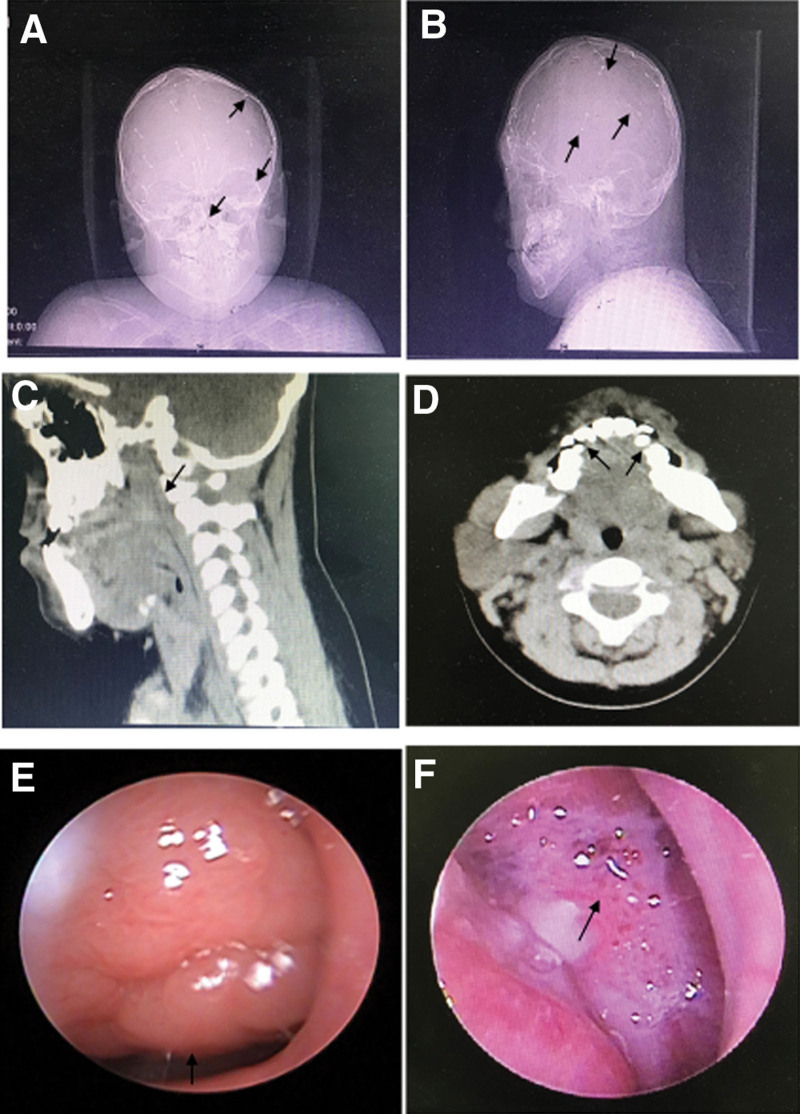
(A, B) Cranial CT showed: frontal-parietal temporal-occipital bone, bilateral orbital lateral wall and nasal bone were unnatural (shown by the arrow → in figure A), multiple metal high-density shadows (shown by the arrow → in figure B) were seen, bilateral eyeballs protruded outwards. (C) Nasopharyngeal CT showed: adenoid hypertrophy, almost completely obstructing the posterior nostril and oppressing the torus tubarius (the arrow → show hypertrophic adenoids); (D) Irregular arrangement of teeth and narrow posterior nasal meatus; (E) Nasal endoscope showed adenoid hypertrophy; (F) Reexamination of nasopharynx showed that the wound of nasopharynx recovered well.

A 9-year-old male patient complained of “Sleep snoring for 6 years, progressive aggravation in the past 1 month and accompanied by apnea during sleep” admitted to the hospital on May 11, 2022. The sleep condition of the child had been worse in recent 1 month, accompanied by obvious snoring after falling asleep, showing typical symptoms of obstructive sleep apnea hypopnea syndrome. He was treated with glucocorticoid and leukotriene receptor antagonist but the effect was not good enough. Crouzon syndrome was diagnosed in Nanjing Children’s Hospital 8 years ago and performed “Fronto-orbito-parietal occipital incision and reconstruction.” Physical examination: frontal bone and mandible protrusion, bilateral exophthalmia, eye distance widened; nose root slightly sunken, shallow boat shape in the middle of the face; tongue hypertrophy, bilateral tonsils not hypertrophy. Cranial CT showed: frontal-parietal temporal-occipital bone, bilateral orbital lateral wall and nasal bone were unnatural, multiple metal high-density shadows were seen, bilateral eyeballs protruded outwards (Fig. 1A and B). Nasopharyngeal CT and nasal endoscopy showed: adenoid hypertrophy, almost completely obstructing the posterior nostril and oppressing the torus tubarius, and tooth arrangement deformity (Fig. 1C–E). Polysomnography results: OAHI = 12.5, LSaO_2_ = 56%. Due to poor cooperation, there was no result of blood gas analysis. Admission diagnosis: 1. Severe obstructive sleep apnea syndrome with severe hypoxemia; 2. Adenoid hypertrophy; 3. Crouzon syndrome. After the preoperative examinations were completed, the multidisciplinary expert cooperation team of our hospital for obstructive sleep apnea conducted a comprehensive evaluation and formulated the treatment plan as follows: the effect of early glucocorticoid and anti-infective treatment was not good, so adenoidectomy could be performed; since the patients had congenital comprehensive malformations, we suggest that noninvasive positive pressure ventilation should be performed before operation to improve the state of hypoxia and be transferred to pediatric intensive care unit (PICU) after operation.

The patient underwent endoscopic hypothermic plasma adenoidectomy under general anesthesia on May 13, 2022. The operation was successful. After operation, the patient was transferred to PICU with oral tracheal intubation and resuscitation sac pressure assisted respiration. On May 16, the tracheal intubation and the ventilator was removed and the nasal catheter was used to inhale oxygen. After removing the ventilator, the blood oxygen decreased, and failed to rise to above 90% after active treatment, and finally re-intubated and ventilator-assisted breathing. On May 21, try to remove the endotracheal intubation and ventilator once again, and the respiratory condition of the child was satisfactory. Continue to maintain anti-infective treatment. The vital signs of the child were stable and he was discharged from hospital on June 10. The reexamination on July 5 showed that the wound of nasopharynx recovered well (Fig. 1F) and the sleep state was significantly improved. He will be reexamined after 6 months.

The report has obtained the informed consent of the patient’s parent (his mother).

## 3. Discussion

Crouzon syndrome is a kind of craniofacial developmental deformity caused by premature closure of cranial suture, which was first reported by French neurologist Octave Crouzon in 1912.^[5]^ This disease is autosomal dominant inheritance, and the mutant gene is located in the gene region of fibroblast growth factor receptor 2 of chromosome 10q25-q26.^[6]^ Fibroblast growth factor receptor 2 belongs to the tyrosine kinase receptor family, but the detail of the mechanism for them is lacking. At present, it is generally believed that gene mutation can excessively activate its downstream signal, thus promoting the differentiation of osteoblasts and leading to premature closure of cranial suture.^[7]^ The order and scope of premature closure of craniofacial suture determine the degree of craniofacial deformity.^[8]^ The earlier the time of premature closure of cranial suture, the greater the influence on the growth and development of children’s skull.^[9]^ The disease can present familial clustering, but there are also about 30% to 60% sporadic case.^[10]^ This case denied the family genetic history and was considered as a sporadic case.

Facial deformity and morphological limitation caused by abnormal bony structure is one of the causes of upper airway collapse. Apnea events occasionally occur during sleep in normal children, with an incidence of about 1% to 3% and the clinical manifestations are mostly alternating between apnea and active inspiration.^[11]^ The abnormal craniofacial morphology of children with craniofacial deformities can cause airflow limitation in any plane of the upper airway, resulting in sleep apnea. The facial outline of children with Crouzon syndrome was sunken, and the nasal cavity was similar to anterior and posterior compression and high arch, which narrowed the posterior nostril and nasopharynx airway. The average nasal resistance was 0.797 kPa·s/L in those children, which was significantly higher than that of normal people,^[12]^ and significantly increased the incidence of sleep apnea. If it is accompanied by adenoid hypertrophy or tonsil hypertrophy, it will further aggravate the development of the disease. An meta-analysis^[13]^ shows that adenoidectomy/tonsillectomy is effective in the treatment of OSA in children with syndromic craniosynostosis. Xue et al^[14]^ reported that 2 children with Crouzon syndrome due to sleep snoring underwent tonsillectomy and adenoidectomy. 1 year after operation, the symptoms including sleep snoring, mouth breathing and holding breath were significantly relieved. Yanyan et al^[10]^ retrospectively analyzed 5 children with Crouzon syndrome all with varying degrees of adenoid hypertrophy. Patients underwent tonsillectomy and/or adenoidectomy and postoperative follow-up showed that the symptoms of sleep snoring were significantly improved in all children. Qiurong et al^[15]^ reported a case of Crouzon syndrome with severe OSAHS and congenital heart disease, sleep snoring and mouth breathing were not found in the 1-year follow-up after adenoidectomy. However, some studies have suggested that for children with craniofacial anatomical abnormalities, adenoidectomy/ tonsillectomy alone can not completely relieve the symptoms of sleep apnea,^[16]^ because there are still high risk factors after operation.^[17]^ As skeletal deformities cannot be resolved, tracheotomy is still unavoidable, accounting for about 20% (47/251).^[18]^ Although the Chinese guideline for the diagnosis and treatment of childhood obstructive sleep apnea (2020) does not provide evidence for surgical intervention in craniofacial morphology, studies had shown that craniofacial surgery can benefit infants, normal children and children with craniofacial deformities. A analysis showed that rapid maxillary dilatation can significantly reduce the index of hypopnea, mandibular distraction osteogenesis can successfully avoid endotracheal intubation in children with failed conservative treatment or as an adjuvant treatment for successful extubation. The operation of posterior cranial distraction osteogenesis is relatively simple, the treatment process is controllable, accurate and stable, and the risk of potential complications is lower, so it can be used as the first choice for the treatment of premature closure of cranial suture syndrome.^[19]^ Frontoorbital advancement and modified Le Fort III distraction osteogenesis are also an alternative reliable surgical procedures. Normalization of the face was obtained in all cases with improvement of the respiratory problems.^[20]^ Combined with the condition of this case, the maxillofacial deformity and tongue hypertrophy led to pharyngeal stenosis, adenoid hyperplasia led to nasopharyngeal stenosis. These upper airway multiplane obstruction caused snoring and mouth breathing during sleep, resulting in long-term chronic hypoxia, further aggravating facial deformities and affecting the growth and intellectual development of the child. Therefore, after multidisciplinary consultation, endoscopic hypothermic plasma adenoidectomy was chosen as the primary choice for relieving airway obstruction.

This patient entered PICU after operation and used ventilator to assist breathing. On the 3rd day after the operation, the patient had difficulty breathing or dyspnea after extubation and intubated again. After analysis, many reasons should be taken into account in this situation. First of all, it may be closely related to multiple inflammation of bilateral lungs, malformation of maxillofacial development and long-term chronic hypoxia. Some studies have suggested that it is difficult to perform extubation after endotracheal intubation or tracheotomy in patients with craniofacial deformities.^[18]^ Secondly, it should also be considered that long-term chronic hypoxia leads to a long-term high level of CO_2_ in the body, which reduces the sensitivity of the respiratory center to the CO_2_ level, and hypoxia had become the main stimulating factor of respiration. After adenoidectomy, upper respiratory tract obstruction had was relieved and high concentration of oxygen was inhaled, which weakens the effect of negative stimulation of respiratory central chemoreceptors, resulting in a decrease in ventilation and an increase in CO_2_ retention, and finally further inhibitis respiration. Therefore, it is necessary to prolong the time of ventilator-assisted breathing and actively conduct anti-infection and fluid replacement therapy. After removing the ventilator, it also should be given non-invasion ventilation by oral-nasal mask, and the respiratory condition and haemogas analysis indicators of the children were closely observed to ensure the stability of vital signs.

Crouzon syndrome is associated with increased perioperative risk due to developmental malformations of the maxillofacial region, abnormal airway structure development, and long-term chronic hypoxia. Therefore, if a patient with Crouzon syndrome need to be treated under general anesthesia, it was necessary to conduct multidisciplinary consultation before operation. Patients should be assessed comprehensively and carefully, and an individual plan should be made according to the specific situation of the patient. After operation, patients should also be closely observed and complications should be treated in a timely manner, so as to reduce the occurrence of perioperative risks. In short, clinicians should be familiar with the clinical manifestations and complications of Crouzon syndrome, diagnose and treat the disease at an early stage, in order to improve the prognosis and life quality of children.

## Acknowledgments

Thanks to everyone who has supported and helped with this research, including other doctors, nurses, and graduate students in our department.

## Author contributions

**Conceptualization:** Yuliang Zhao.

**Data curation:** Lei Yu.

**Writing – original draft:** Lei Yu, Yuliang Zhao.

**Writing – review & editing:** Lei Yu, Yuliang Zhao.
